# Supplementary material for: Career self-efficacy disparities in underrepresented biomedical scientist trainees
Source: PLoS One. 2023 Mar 1;18(3):e0280608. doi: 10.1371/journal.pone.0280608 (PMC9977038; doi:10.1371/journal.pone.0280608)
Supplement: S1 File — (PDF) [file pone.0280608.s001.pdf]

S1. Abbreviations

ANOVA = Analysis of variance
CIMER = Center for the Improvement of Mentored Experience in Research
IMSD = Initiative for Maximizing Student Development
IRB = institutional review board
IRACDA = Institutional Research and Academic Career Award Program
MARC = Maximizing Access to Research Careers
NIH BEST = National Institutes of Health Broadening Experiences in Scientific Training award
PI = Principal Investigator (research-intensive)
RISE = Research on Improving Systems of Education
SCCT = Social Cognitive Career Theory
STAR = Stipend for Training Aspiring Researchers
STEM =Science Technology Engineering and Math
UR = Underrepresented
WR = Well-Represented
US = United States of America
