# Supplementary material for: Career self-efficacy disparities in underrepresented biomedical scientist trainees
Source: PLoS One. 2023 Mar 1;18(3):e0280608. doi: 10.1371/journal.pone.0280608 (PMC9977038; doi:10.1371/journal.pone.0280608)
Supplement: S2 File — (PDF) [file pone.0280608.s002.pdf]

S2 – Supplemental Table 1. Questionnaire Items

What year are you in your Ph.D. program?	1=1st year<br>2=2nd year<br>3=3rd year<br>4=4th year<br>5=5th year<br>6=6th year or more
Which of the following best describes your ethnicity? *(optional)*	1=Hispanic or Latino<br>2=Not Hispanic or Latino
Q35. Which of the following describes your race? (Please check all that apply) *(optional)*	
a. American Indian or Alaska Native	1=Checked<br>0=Not checked
b. Asian	1=Checked<br>0=Not checked
c. Black or African American	1=Checked<br>0=Not checked
d. Native Hawaiian or other Pacific Islander	1=Checked<br>0=Not checked
e. White	1=Checked<br>0=Not checked
What is your citizenship status? *(optional)*	1=US citizen since birth<br>2=Naturalized US citizen<br>3=Non-US citizen with permanent resident visa (green card)<br>4=Non-US citizen with temporary US visa
What is your gender? *(optional)*	1=Male<br>2=Female

How confident are you that you can do the following?<br><br>1=Not at all confident<br>2=Minimally confident<br>3=Moderately confident<br>4=Highly confident<br>5=Completely confident

1. Assess your abilities to pursue your desired career path(s)
2. Determine the steps to pursue your desired career path(s)
3. Seek advice from professionals in your desired career path(s)
4. Identify potential employers, firms, and institutions relevant to your desired career path(s)
5. Achieve your career goals
6. How confident are you to discuss your career goals with your PI/thesis advisor?

Q5. Based on the list of career paths, to what extent are you currently considering the following?<br><br>0=Not familiar enough to decide<br>1=Not at all considering<br>2=Slightly considering<br>3=Moderately considering<br>4=Strongly considering<br>5=Will definitely pursue
a. Principal investigator in a research-intensive institution
b. Research in industry
c. Research staff in a research-intensive institution
d. Combined research and teaching careers
e. Teaching-intensive careers in academia
f. Science education for K-12 schools
g. Science education for non-scientists
h. Clinical practice
i. Public health related careers
j. Scientific/medical testing
k. Science writing
l. Research administration
m. Science policy
n. Intellectual property
o. Business of science
p. Entrepreneurship
q. Sales and marketing of science-related products
r. Support of science-related products
s. Drug/device approval and production
t. Clinical research management
u. Career that is not related to science (please specify): _______________
