# Supplementary material for: Career self-efficacy disparities in underrepresented biomedical scientist trainees
Source: PLoS One. 2023 Mar 1;18(3):e0280608. doi: 10.1371/journal.pone.0280608 (PMC9977038; doi:10.1371/journal.pone.0280608)
Supplement: S3 File — (PDF) [file pone.0280608.s003.pdf]

S3 - Supplemental Table 2. Inter-reliability of Items ($\alpha = 0.86$)

Deleted Variable	Item Correlation with Total	Alpha
Item 1. Self-Assess abilities to pursue desired…	0.72	0.83
Item 2. Determine the steps to pursue desired…	0.73	0.83
Item 3. Seek advice from professionals in desired…	0.66	0.84
Item 4. Identify potential employers/institution…	0.67	0.84
Item 5. Achieve career goals	0.65	0.85
