# Supplementary material for: Career self-efficacy disparities in underrepresented biomedical scientist trainees
Source: PLoS One. 2023 Mar 1;18(3):e0280608. doi: 10.1371/journal.pone.0280608 (PMC9977038; doi:10.1371/journal.pone.0280608)
Supplement: S4 File — (PDF) [file pone.0280608.s004.pdf]

S4 – Supplemental File. Interactive Data Set Availability

Data have been aggregated into the categories identified in the *Methods* section to protect identities of subjects (e.g., Race/Ethnicity aggregated to Underrepresented). The interactive dataset is available via Tableau, such that readers can isolate the factors of interest and examine effects on career self-efficacy by any combination of factors combined or independently (e.g., Gender, Race/Ethnicity, Career Interest, Seniority). Note that HTML code can be provided to host on PLOS ONE if desired. (Link: https://public.tableau.com/app/profile/jan1760/viz/06152022_Careerselfefficacy_Interactionsofvariables/Interactionpatterns).
