# Supplementary material for: Career self-efficacy disparities in underrepresented biomedical scientist trainees
Source: PLoS One. 2023 Mar 1;18(3):e0280608. doi: 10.1371/journal.pone.0280608 (PMC9977038; doi:10.1371/journal.pone.0280608)
Supplement: S5 File — (PDF) [file pone.0280608.s005.pdf]

S5. Supplemental Table 3. Tukey's corrected multiple comparisons for Gender, Race/Ethnicity, Career Interest, and Seniority

Tukey's multiple comparisons test	Mean Diff.	95% CI of diff.	Sig	Adj P-Value
Male WR Non-PI Junior vs. Male WR PI Junior	-0.29	-0.43 to -0.15	****	<0.0001
Male WR Non-PI Junior vs. Male WR PI Senior	-0.22	-0.37 to -0.06	***	0.0002
Male WR Non-PI Junior vs. Male UR PI Junior	-0.38	-0.63 to -0.12	****	<0.0001
Male WR Non-PI Junior vs. Male UR PI Senior	-0.37	-0.70 to -0.04	*	0.0101
Male WR Non-PI Junior vs. Female WR PI Junior	-0.21	-0.35 to -0.07	****	<0.0001
Male WR Non-PI Junior vs. Female WR PI Senior	-0.24	-0.39 to -0.08	****	<0.0001
Male WR Non-PI Junior vs. Female UR PI Junior	-0.27	-0.51 to -0.02	*	0.0185
Male WR Non-PI Senior vs. Male WR PI Junior	-0.33	-0.47 to -0.18	****	<0.0001
Male WR Non-PI Senior vs. Male WR PI Senior	-0.26	-0.42 to -0.10	****	<0.0001
Male WR Non-PI Senior vs. Male UR PI Junior	-0.41	-0.68 to -0.15	****	<0.0001
Male WR Non-PI Senior vs. Male UR PI Senior	-0.41	-0.74 to -0.08	**	0.0022
Male WR Non-PI Senior vs. Female WR PI Junior	-0.25	-0.39 to -0.10	****	<0.0001
Male WR Non-PI Senior vs. Female WR PI Senior	-0.27	-0.43 to -0.11	****	<0.0001
Male WR Non-PI Senior vs. Female UR PI Junior	-0.30	-0.55 to -0.06	**	0.0027
Male WR PI Junior vs. Female WR Non-PI Junior	0.36	0.24 to 0.49	****	<0.0001
Male WR PI Junior vs. Female WR Non-PI Senior	0.34	0.21 to 0.47	****	<0.0001
Male WR PI Junior vs. Female UR Non-PI Junior	0.46	0.26 to 0.66	****	<0.0001
Male WR PI Senior vs. Female WR Non-PI Junior	0.29	0.15 to 0.43	****	<0.0001
Male WR PI Senior vs. Female WR Non-PI Senior	0.27	0.12 to 0.42	****	<0.0001
Male WR PI Senior vs. Female UR Non-PI Junior	0.39	0.18 to 0.60	****	<0.0001
Male UR Non-PI Senior vs. Female WR Non-PI Junior	0.32	0.01 to 0.63	*	0.0395
Male UR Non-PI Senior vs. Female UR Non-PI Junior	0.41	0.06 to 0.76	**	0.0052
Male UR PI Junior vs. Female WR Non-PI Junior	0.45	0.20 to 0.70	****	<0.0001
Male UR PI Junior vs. Female WR Non-PI Senior	0.42	0.17 to 0.68	****	<0.0001
Male UR PI Junior vs. Female UR Non-PI Junior	0.54	0.25 to 0.84	****	<0.0001
Male UR PI Senior vs. Female WR Non-PI Junior	0.45	0.13 to 0.77	***	0.0002
Male UR PI Senior vs. Female WR Non-PI Senior	0.42	0.10 to 0.75	***	0.0009
Male UR PI Senior vs. Female UR Non-PI Junior	0.54	0.18 to 0.90	****	<0.0001
Female WR Non-PI Junior vs. Female WR PI Junior	-0.28	-0.41 to -0.16	****	<0.0001
Female WR Non-PI Junior vs. Female WR PI Senior	-0.31	-0.45 to -0.17	****	<0.0001
Female WR Non-PI Junior vs. Female UR Non-PI Senior	-0.24	-0.47 to -0.01	*	0.0245
Female WR Non-PI Junior vs. Female UR PI Junior	-0.34	-0.58 to -0.10	****	<0.0001
Female WR Non-PI Junior vs. Female UR PI Senior	-0.30	-0.60 to -0.00	*	0.043

Female WR Non-PI Senior vs. Female WR PI Junior	-0.26	-0.39 to -0.13	****	<0.0001
Female WR Non-PI Senior vs. Female WR PI Senior	-0.29	-0.43 to -0.14	****	<0.0001
Female WR Non-PI Senior vs. Female UR PI Junior	-0.32	-0.56 to -0.08	***	0.0006
Female WR PI Junior vs. Female UR Non-PI Junior	0.37	0.18 to 0.57	****	<0.0001
Female WR PI Senior vs. Female UR Non-PI Junior	0.40	0.19 to 0.61	****	<0.0001
Female UR Non-PI Junior vs. Female UR Non-PI Senior	-0.34	-0.61 to -0.06	**	0.0031
Female UR Non-PI Junior vs. Female UR PI Junior	-0.43	-0.72 to -0.15	****	<0.0001
Female UR Non-PI Junior vs. Female UR PI Senior	-0.39	-0.73 to -0.06	**	0.0055

Supplemental Table 2 Legend. Tukey’s adjusted multiple comparisons for Gender, Race/Ethnicity, Career Interest, and Seniority. P-values indicate significance analyzed by Tukey's multiple comparisons tests, ****p<0.0001, ***p<0.001, **p<0.01, and *p<0.05.
